# High frequency headache prevalence and management in primary care. A survey among general practitioners of the Liege area, Belgium

**DOI:** 10.1186/1129-2377-1-S14-P218

**Published:** 2013-02-21

**Authors:** D Magis, J Schoenen

**Affiliations:** 1 Headache Research Unit - University Department of Neurology, Liege, Belgium

## Introduction

Headache is among the most frequent neurologic symptoms that lead patients to consult a general practitioner (GP). High frequency headaches (HFH) are very disabling and their management can be a real challenge even for a neurologist, which is likely due to CNS changes occurring with chronic headache and to frequent comorbidities. HFH patients need a multimodal therapeutic approach that is quasi non-existing in Belgium.

## Objectives

The aim of this survey was to determine the prevalence and management of patients with HFH (min 2 headache days/week) in a cohort of general practitioners in order to evaluate the need and necessary resources for a 'Centre for Integrated Multimodal Treatment of Chronic Headaches' (CIMIC) in Liege.

## Methods

A short questionnaire (10 questions) was distributed to 250 GPs of the Liege area (population: 616 491) with the help of the representatives of a pharmaceutical company.

## Results

The responder rate was 26% only (N=65). In the last 2 months most GPs examined 1 to 50 patients with HFH. Eighty percent of these HFH patients were also followed by a neurologist, 16.9% consulted a headache specialist and 23% a pain clinic. The majority of GPs (86.1%) reported that at least 50% of their HFH patients had acute medication overuse (>2 tablets/week). About 50% of HFH patients were considered by their GP to be depressed but a minority had other chronic pain disorders. Interestingly, few patients (0-25% on average) had a multimodal headache management (for example a preventive drug treatment combined with behavioural therapy) and most HFH patients were dissatisfied with their current headache management.

## Conclusions

This short survey among GPs practicing in the Liege area confirms that high frequency headache patients are not rare and in need of improved management, in spite of the fact that many of them are followed by a neurologist. Indeed, most of them are dissatisfied with current care, have acute drug overuse (which is a known aggravating factor for headaches), and are depressed. These patients are likely to benefit from integrated care with multimodal management in a centre like CIMIC. Unfortunately, the low participation rate might reflect the lack of interest and/or time GPs have for chronic headache sufferers. Acknowledgements The authors thank the medical representatives of Grunenthal for the delivery of the questionnaires to GPs.

